# From Soil Amendments to Controlling Autophagy: Supporting Plant Metabolism under Conditions of Water Shortage and Salinity

**DOI:** 10.3390/plants11131654

**Published:** 2022-06-22

**Authors:** Hans-Werner Koyro, Bernhard Huchzermeyer

**Affiliations:** 1 Institute of Plantecology, Justus-Liebig-University, Heinrich-Buff-Ring 26, 35392 Giessen, Germany; 2 Institute of Botany, Leibniz Universitaet Hannover, Herrenhaeuser Str. 2, 30416 Hannover, Germany; bernhard.huchzermeyer@gmx.de or; 3 AK Biotechnology, VDI-BV-Hannover, Hanomagstr. 12, 30449 Hannover, Germany

**Keywords:** water withhold, salinity, stress amendments, biochar, plant–microorganism interaction, plant growth promoting bacteria (PGPB), hormone, auxin, ethylene, autophagy, stress perception and signaling

## Abstract

Crop resistance to environmental stress is a major issue. The globally increasing land degradation and desertification enhance the demand on management practices to balance both food and environmental objectives, including strategies that tighten nutrient cycles and maintain yields. Agriculture needs to provide, among other things, future additional ecosystem services, such as water quantity and quality, runoff control, soil fertility maintenance, carbon storage, climate regulation, and biodiversity. Numerous research projects have focused on the food–soil–climate nexus, and results were summarized in several reviews during the last decades. Based on this impressive piece of information, we have selected only a few aspects with the intention of studying plant–soil interactions and methods for optimization. In the short term, the use of soil amendments is currently attracting great interest to cover the current demand in agriculture. We will discuss the impact of biochar at water shortage, and plant growth promoting bacteria (PGPB) at improving nutrient supply to plants. In this review, our focus is on the interplay of both soil amendments on primary reactions of photosynthesis, plant growth conditions, and signaling during adaptation to environmental stress. Moreover, we aim at providing a general overview of how dehydration and salinity affect signaling in cells. With the use of the example of abscisic acid (ABA) and ethylene, we discuss the effects that can be observed when biochar and PGPB are used in the presence of stress. The stress response of plants is a multifactorial trait. Nevertheless, we will show that plants follow a general concept to adapt to unfavorable environmental conditions in the short and long term. However, plant species differ in the upper and lower regulatory limits of gene expression. Therefore, the presented data may help in the identification of traits for future breeding of stress-resistant crops. One target for breeding could be the removal and efficient recycling of damaged as well as needless compounds and structures. Furthermore, in this context, we will show that autophagy can be a useful goal of breeding measures, since the recycling of building blocks helps the cells to overcome a period of imbalanced substrate supply during stress adjustment.

## 1. Introduction

The continuous growth of human population is a major threat to global food security. The situation is worsening with the increasing demand for biomass as raw material for industrial use. Therefore, food production and industrial interests are competing for resources as can be observed, for instance, in the case of the food–feed–fuel competition [1]. Global scarcity of water resources, increased environmental pollution, and salinization of soil and fresh water are adding to this problem [2]. In addition, we have to consider that global climate change will increase the probability of the occurrence of extreme environmental situations. Among these, high winds, extreme temperatures, drought, and flood are reported. In their study, Daryanto et al. (2016) [3] have analyzed information on crop yield, which was published from 1980 to 2015. Worldwide, water limitation has caused up to 21 and 40% yield reductions in wheat and maize, respectively. Moreover, soil salinity results in major reductions in cultivatable land area, and decreases crop productivity and quality. This is particularly the case in salt-affected farmland in the arid irrigation area, with an approximate rate of around 1–2% annually [4]. By 2050, it is estimated that 50% of all arable land will be impacted by salinity [5]. Currently, almost 1 billion ha of arable land is affected by salinity, representing about 7% of the Earth’s continental area [5]. In addition, reduced productivity is causing more than USD 12 billion in annual losses [6,7]. Global food production is required to double by the year 2050 to meet the ever-growing demands of the population [8]. Therefore, understanding the mechanisms underlying plant abiotic stress responses and the generation of plants that are resistant to environmental stress has received considerable attention in recent years [9]. Stress-resistant plants can initiate a variety of changes at the molecular, cellular, and physiological traits as well as signal transduction pathways, to survive under adverse environmental conditions [10,11]. In this context, we have to separate between short-term heatwaves or heavy rainfall and long-term events extending over periods of several weeks [12]. In particular, these long lasting events will affect the environment as a whole, and in terms of agricultural land use, will require the respective adaptation of cropping. With recent progress in advanced genomics and high-throughput sequence technologies, genes involved in many of the essential steps that regulate the molecular mechanism as well as stress-responsive genes have been identified and characterized. The identification of stress signaling molecules, transduction pathways, and discovery of ABA receptors have significantly improved our understanding of functions as well as transcriptional and post-transcriptional regulation of stress-responsive gene expression [13].

However, plant performance can be improved under adverse environmental conditions by integrated soil–water–plant solutions or integrated soil fertility and plant nutrient management [14]. A great challenge for agriculture is land degradation, along with a reduction in soil fertility and organic and inorganic soil resources for sustainable crop and livestock production.

In this review, we will focus on drought and salinity stress with respect to two aspects: (i) The improvement of growth conditions by soil amendments, such as biochar, and (ii) inoculation by beneficial microorganisms. With the use of microbes and biochar, soil fertility can remain and soil organic resource can be recycled back to the farm. We will describe the observed beneficial effects, and try to explain the physiological and biochemical reactions underlying the observed modifications of plant performance in a stressful environment. In the context of the observed stress response, we will discuss the function and regulation of autophagy. To be more specific, we will try to establish to what extent providing building blocks for recycling can improve the stress resistance of plants. Even though a detailed analysis of the beneficial effects of plants and microorganisms has not been performed at the molecular level to date, we will include this topic in this review. This may allow us to identify the parameters that indicate the degree of stress resistance and to discuss future strategies that lead to an improved stress response of crops.

## 2. Plant Performance in the Field

Plants are sessile organisms. In the field, they have to cope with some fluctuating environmental factors. Therefore, plants have a certain window of resistance that allows for instant adaptation to the respective spectrum of environmental factors [15], with different resistances among plant species. In several experiments, it was observed that a narrow tolerance window correlates with a high growth rate, while broadening the window of resistance will result in reduced growth rates [16,17,18]. Therefore, it may be expected that at locations with only little fluctuation in environmental factors, and rare incidents of extreme weather conditions, the most abundant plant species may be characterized with a narrow window of resistance [19]. Several of our crops are characterized with a narrow window of resistance as compared with their wild ancestors, since breeding programs were aimed at high-yielding crops. In contrast to earlier breeding concepts, more recent projects are aimed at breeding for crop accessions that will produce a reliable yield, even under slightly suboptimal growth conditions [20,21,22,23,24].

Fluctuating illumination on a cloudy day, day–night changes, etc. are environmental changes that each plant has to tolerate. Plants will preferentially adapt by the regulation of enzyme activities rather than the production of new enzymes and modification of enzyme abundance. Therefore, these responses can occur almost instantly and easily escape detection in the field. Accordingly, most of the experiments that analyze these immediate responses were carried out in laboratories [25]. In general, these short-term events will not cause immediate damage, but may affect the resource use efficiency. On the other hand, long lasting periods of drought, heat, lack of nutrients, soil salinization, etc., will adversely affect plant performance at a significant degree. These conditions have been termed as persistent stress. In accordance with Larcher [26], we have to distinguish between (i) mild stress, which allows stressed plants to thrive and subsequently re-adjust to a new physiological equilibrium, and (ii) severe, lethal stress, which is beyond the resistance level of the respective plant species (see Table 1).

In the literature, environmental stress is often referred to as abiotic stress to differentiate it from biotic stress, which is caused by pathogens and herbivores. With respect to its global economic importance, abiotic stress is estimated to cause 50 to 80% of yield losses in crop production [27,28]. The calculation is based on a comparison between the current annual crop yield and theoretical yield. This can be performed by scientists under completely controlled conditions in a greenhouse [29]. Therefore, the precise extent of yield reduction can be questioned. Nevertheless, the enormous economic impact of abiotic stress is beyond any discussion.

Plant response to abiotic stress is described as a multifactorial trait [30]. Moreover, plant species differ to a large degree in the preferential use of response patterns. This applies to any type of abiotic stress. Drought and salt stress are of primary importance for agriculture in arid areas. However, these two stresses can be found in other climatic zones, as well. Accordingly, many scientists have focused on analyzing the effects induced by the application of these two types of stress. The common trait between both stresses is that plant roots can sense low levels of available water. Therefore, some of the plant responses to these two types of abiotic stress are quite similar, such as osmolyte production (sugars, amino acids, and other organic molecules), in which the osmotic potential in plant roots adjusts to facilitate water uptake from the soil. In the case of soil salinity, some plants use an import of inorganic ions rather than osmolyte synthesis (on the expense of assimilate consumption) to control cellular osmotic potential [31,32,33].

**Table 1:** Plant response to environmental stress.

Type of Stress	Application/Duration	Example	Reference
Eustress	whole plant priming	taking advantage of cross tolerance to different types of stress	Villagómez-Arande et al., 2022 [34]
Distress	fluctuating environmental conditions	day–night cycles; rain–sunshine cycles	Lichtenthaler, 1996 [15]
	short time, hours to 4 days	laboratory experiments to identify stress-responsive traits (genes)	Miller et al., 2015 [35]
	series of adverse environmental conditions (weeks to months)	drought periods	Fahad et al., 2017 [36]
	poor growth conditions	soil salinity lack of nutrients deserts	Zhao et al., 2020 [37]
	transfer to a different environment	cultivation of plants not native to an area	Geppert et al., 2021 [38]

**Figure 1:**
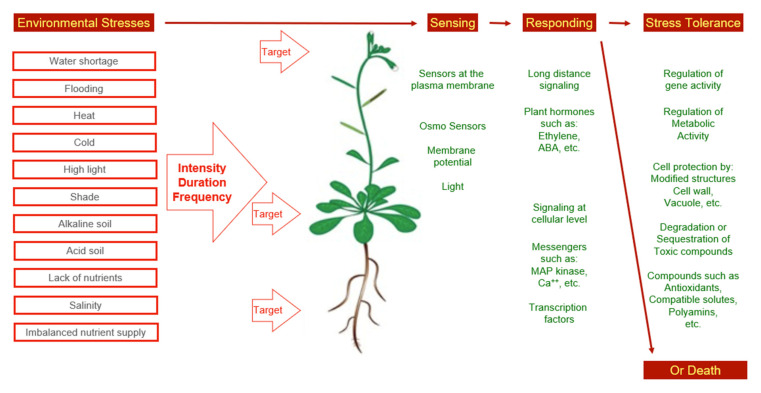
Environmental stress and observed concepts of stress adaptation.

All types of stress will affect the physiology of a plant as a whole. This indicates that stress perception is followed by signaling events (Figure 1). In the case of water shortage and salinity, ABA is one of the messengers. However, reactions other than closure of stomata and abscission of leaves can take place as a response to abiotic stress, as well. Among the stress responses, major physiological processes not under ABA control, such as nitrogen fixation, respiration, photosynthesis, and carbohydrate metabolism are included [39].

From the description of ecosystems, it was evident that plant species differ to a large degree with respect to environmental preferences. In addition, this applies to the stress resistance level of plants. For instance, a puzzling observation was subsequent salinization, in which a new plant population occupied abandoned areas. These highly salt-resistant species are referred to as halophytes [40,41,42]. Respective plant species turned into experimental plants in many research teams. The idea was to compare sensitive and resistant plant species to better understand concepts of stress perception, signaling, and processing. This approach requires the standardization of methods to allow for a comparison of the results. Moreover, this need was addressed in a handbook that provides standardized protocols for the measurement of plant features, which reflect the ecological strategies of species [43]. After 10 years, the handbook has been updated to provide protocols on traits and methods that had proven to be useful in laboratories as well as in field studies. Furthermore, the new handbook contains additional protocols for functional traits of organs apart from leaves [44].

The traits described in the two handbooks were selected to predict long lasting effects at the ecosystem level. In addition, they might be used for prediction of annual crop yield, although remote sensing methods are preferentially used for these aspects. Moreover, the described standardized methods can be used to monitor the success of stress resistance breeding. However, they do not provide direct information on potential breeding targets at the metabolic and molecular level. Therefore, an impressive number of publications on genome, proteome, and metabolome analyses of plant species and accessions that differ in their stress resistance are available in the current literature [45,46,47]. Regulation of gene expression during the phase of stress adaptation has been analyzed, and the importance of individual genes for expression of a certain resistance feature has been analyzed by targeted mutation of experimental plants [48,49,50].

This was very helpful in the study of stress response, in which significant differences could be detected in accessions of the same plant species when harvested from areas with different local climates, soil conditions or the availability of water [51]. However, even when using plants as different as Arabidopsis and maize, differences in chromatin modification have been found [52,53].

Notably, the term stress does not exclude positive implications. Moderate stimuli have a positive impact (eustress), whereas excessive stimuli have a negative impact (distress) on plant response. Moreover, the positive effect of extra compounds as different as sugars, amines, elevated CO_2_ concentration, hormones, H_2_O_2_, UV radiation or nanomaterial has been tested in a concentration-dependent manner [54,55,56,57,58,59]. Several authors reported that a repetitive application of moderate stress (with or without the addition of compounds) can modify metabolism, fluidity of biomembranes, the content of compatible solutes and ROS scavenging antioxidants, and enzymes [60]. Furthermore, the parameter value of the stress resistance window of treated plants correlated with the content of these beneficial compounds. Based on these observations, it is a matter of ongoing discussion, whether improved plant performance under stress in the presence of symbionts relies on similar regulatory mechanisms. In the meantime, economic interest has dramatically increased by the fact that (i) metabolites of pharmaceutical interest were among the compounds overproduced under eustress, and (ii) green algae and higher plants responded similarly to the applied eustress [61]. We will refer to these aspects in the following section.

## 3. Photosynthetic Performance under Stress

Primarily, it is an astonishing observation that very different types of stress lead to an impairment of photosynthesis. However, there is evident commonality, in terms of an inhibition in the growth and development of plants. In the following overview of photosynthesis, we would like to show how the stress-related inhibition of photosynthetic reactions can be derived from this connection. In particular, enzyme-kinetic considerations explain why ROS increases under stress, related to the observed damage of leaves.

**Figure 2:**
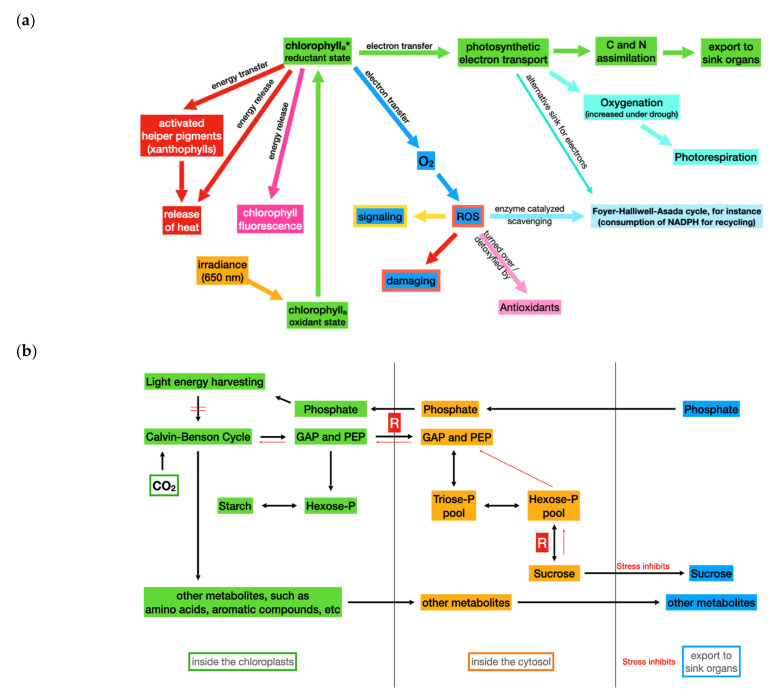
Light energy capture in photosynthesis. (**a**) Pathways competing for absorbed light energy. (**b**) Assimilated export of the chloroplasts via the cytosol to sink tissues. * indicates the light activated state of chlorophyll a.

Moreover, it is evident that plants are dependent on the supply of light energy, although at varying intensities and spectral qualities. For instance, few plant species are known to preferentially thrive in the shade of other plants inside a forest, while other plants grow in full sunshine in the field. At both locations, plants have to tolerate fluctuating light intensities in a certain bandwidth. However, the photosynthetic activity will significantly decrease as compared to control plants, if the applied light intensity will exceed a critical threshold for a prolonged period of time. Adverse effects related to this symptom were summarized as photoinhibition [62]. Furthermore, it was documented that during photoinhibition, the ROS mediated bleaching of pigments follows an initial phase of reduced assimilatory activity [63]. Surplus light energy initially results in quenching of variable chlorophyll fluorescence, resulting from increased thermal dissipation of excitation energy [64]. Subsequently, for instance, the rate of chloroplast protein degradation of the D1 protein of photosystem II exceeds the rate of de novo synthesis [65]. This results in structural changes in the reaction centers of photosystem I and II, a reduced light use efficiency, a reduced photosynthetic electron transport rate, and subsequent assimilation [62]. The initial effects of photoinhibition are reversible if the period of excess light does not last for a long period of time. The mechanisms that lead to increased tolerance to high light intensity are based on a controlled release of surplus energy in the form of heat [66]. An overview of the reactions that compete for absorbed light energy is provided in Figure 2a, the regulation of assimilate export from the source to the sink tissues in Figure 2b.

The redox potential of activated chlorophyll allows for two electron transport pathways: (i) Electron transfer to molecular oxygen (formation of ROS), and (ii) reduction of a component of the photosynthetic electron transport chain. If the assimilation rate is limited and the energy accepting coenzymes, ADP and NADP^+^, will not be recycled, the probability of ROS production will be increased.

The fluorescence energy can be released at a low probability. Since all pathways are competing for energy from the activated chlorophyll, any alternative pathway will result in a reduction of chlorophyll fluorescence. This can be measured as fluorescence quenching.

ROS formation and photosynthetic electron transport are competing for electrons from the light-activated chlorophyll. If assimilation is inhibited under stress, ADP and NADP^+^ will not be recycled. As a result, the rate of ROS formation can exceed the detoxication capacity of chloroplasts.

The Foyer–Halliwell–Asada cycle is most efficient in the removal of ROS [67]. As a side effect, it serves as an alternative sink for electrons, since they are used to regenerate its substrate ascorbate.

When stress results in the closure of stomata and the O_2_/CO_2_ ratio is increasing, there is an increasing probability of 2-PGA production by the oxygenase activity of rubisco. The 2-PGA will be removed by the intrinsic photo-respiration activity of leaf cells. Due to its energy demand and the release of CO_2_, photorespiration contributes to the prevention of ROS production. These reactions contribute to the parameter “photosynthetic quenching” when evaluating stress effects through the monitoring of chlorophyll fluorescence.

The phloem transport of assimilates depends on the availability of water. In addition, the consumption of assimilates by storage organs, meristems, etc. is an important driver of the export of assimilates from source tissues. There are carbohydrate pools in the cytosol and the chloroplast stroma, but the buffer capacity is limited (see Figure 2b). Moreover, carbohydrate export of the chloroplasts is regulated through the inhibition of feedback when the cytosolic hexose pool is filled. Rate limiting steps in carbohydrate transport and conversion are indicated by “R” and stress effects are indicated in red, as shown in Figure 2.

The availability of inorganic phosphate is a second important regulator. The export of triose phosphate from leaf chloroplasts is strictly coupled to an import of phosphate. Therefore, the lack of phosphate as well as the increasing cytosolic hexose concentration will inhibit the assimilation rate [68,69,70].

If the absorption rate of light quanta is exceeding the rate of electron use by NADP^+^ production and subsequent assimilation, an overreduction of the photosynthetic electron transport chain will occur [71]. This can result in the production of reactive oxygen species (ROS). Light-activated chlorophyll as well as reduced ferredoxin are strong electron donors. The negative redox potential allows for the transfer of an electron to molecular oxygen, thus generating a radical [72]. Chloroplasts are equipped with antioxidants as well as ROS detoxification enzyme catalyzing sequences. However, depending on the plant species and the developmental stage of plants, the rate of ROS production may not exceed a specific threshold value [73]. If the rate of ROS production exceeds the rate of its removal, ROS concentration will increase and may reach toxic levels [74].

One strategy for increasing stress resistance is the development of a high capacity system to remove ROS. An alternative strategy is the prevention of ROS production. During evolution, “safety valves” have been developed to accomplish the following: (i) Compete with CO_2_ assimilation for electrons (ii) or remove or degrade assimilates. The most efficient examples are the water–water cycle [75] and photorespiration [76,77,78], as shown in Figure 2a. These reaction sequences were found to be active in parallel to “regular” photosynthesis. Therefore, futile sequences and assimilation are competing for absorbed light energy [79]. As a result, it was assumed that the individual regulation of these sequences may be the basis for individual stress resistance of plant species [80,81,82]. However, stress resistance is achieved at the expense of light energy use efficiency. For this reason, breeding for high-yielding crop species was suggested by reducing or even eliminating these futile sequences [83,84]. In this context, the following questions are addressed: (i) How can the respective activities be measured, and (ii) how can sensors and messengers be identified, i.e., the mechanism controlling futile sequences [85,86,87,88,89]. However, even the general feasibility of this idea was doubted, since photorespiration is an essential pathway in C3 plants and important for the regulation of reactive oxygen species (ROS) [90,91,92,93]. Indeed, it has been proposed that high rates of photorespiration under environmental stresses (e.g., drought stress) can serve an important photoprotective role by maintaining electron acceptor sinks [90]. When the NADP^+^/ATP ratio is high (i.e., due to a high ATP demand resulting from high temperature or water stress), a high electron pressure occurs on PSI acceptors [94]. This pressure and surplus of energy is reduced by photorespiration, since this pathway requires energy (ATP) and reducing NADP^+^ equivalents as well as releases CO_2_ that can be refixed. In addition to the protection support from photoinhibition, photorespiration removes toxic metabolic intermediates, supports plant defense reactions, and is intimately integrated in primary metabolism.

In recent years, several pieces of information have accumulated, which indicate that light-induced ROS release from chloroplasts plays a key role in retrograde signaling to the nucleus [87,88,89,90,91,92,93,94,95,96,97,98]. These data have been arranged in a general signaling network, which is documented in a review by Leister [99].

In many publications on stress responses, the following question was addressed: What is the reason for the over-production of ROS as a common response to different stress events, such as water shortage, high light intensity, heavy metal contamination, etc. Moreover, what these stresses have in common is that the export of assimilates and assimilate usage in sink organs are inhibited. Therefore, increased assimilate concentration in leaf tissues will result in an inhibited carbohydrate export from the chloroplasts [100,101,102]. Inhibition of the Calvin–Benson cycle will inhibit the consumption of NADP^+^ and ATP. Both coenzymes will not be recycled to function as primary acceptors of photosynthetic energy flow. The concentration of the chloroplastic pools of these two coenzymes is extremely low (about 1 mM) [103,104]. Therefore, functioning of the photosynthetic electron transport and reduction of ROS production depends on a permanent recycling of the two energy acceptors NADP^+^ and ADP [68,70,71,80] (see Figure 2b for an overview).

As outlined above, the common sequence of response to drought stress is through the closure of stomata, reduction of gas exchange and transpiration rate, and increase in the O_2_/CO_2_ concentration ratio inside the leaves, thus, enhancing the probability that photorespiration will occur. As a result, in most crops (C3 plants), an increase in the light compensation point was found to be correlated with the intensity of applied drought stress [81]. However, this correlation was observed as less significant or not detectable in C4 plants, such as maize and sorghum [105,106]. Moreover, this observation initiated research activities that are aimed at a transfer of the C4 status to C3 crops, such as rice [107,108,109]. The idea is to take advantage of the fact that in C4 and CAM plants the rubisco activity is restricted to the bundle sheath chloroplasts, a compartment containing a very low O_2_/CO_2_ ratio, thus eliminating the probability of performing photorespiration [110,111]. This approach requires a detailed understanding of metabolite flux between the bundle sheath and the surrounding leaf mesophyll [112]. Furthermore, this knowledge could be used to improve the performance of C4 plants, since the bottlenecks that limit the intermediate transfer between the bundle sheath and mesophyll are only poorly understood [113,114]. Even more demanding is the identification of sensors and messengers involved in the control of the C4 metabolism as well as the regulation of gene activities, resulting in the C4 leaf anatomy [81,115].

## 4. Improving Soil Quality by the Addition of Biochar

Plant performance can be improved under adverse environmental conditions by the addition of soil amendments, such as biochar. The beneficial effect of biochar on soil quality has been tested using experimental plants as different as *solanaceae* (tobacco) [116], *fabaceae* (beans) [117], and *poaceae* (phragmites) [118]. Stimulation of plant biomass production by the addition of biochar was most significant when using marginal soil as a matrix. In the case that the compost was added along with the biochar, the positive effect became even more impressive. However, it was very evident that the type of compost had to be carefully selected, considering the requirements of the tested plant species and the composition (eventual contaminations and mineral content) of the soil [116,117]. The same observation holds true with respect to different types of biochar. Different types of biochar significantly differed in the extent of induced enhancements [119]. Biochar contains a variety of beneficial compounds that are released from the biochar matrix at different rates [120]. Success of biochar amendment was monitored by measuring the soil parameters as well as the physiological and biochemical traits of experimental plants. With respect to soil parameter, it was found that the positive effect of biochar correlated with the relative increase in the organic carbon content of soil. Moreover, it was measured that biochar enhanced the water binding capacity, thus the effect of drought spells was minimized. The same holds true for a better nutrient binding capacity, which provides a continuous release of nutrient ions to the soil and water phase [120,121].

**Figure 3:**
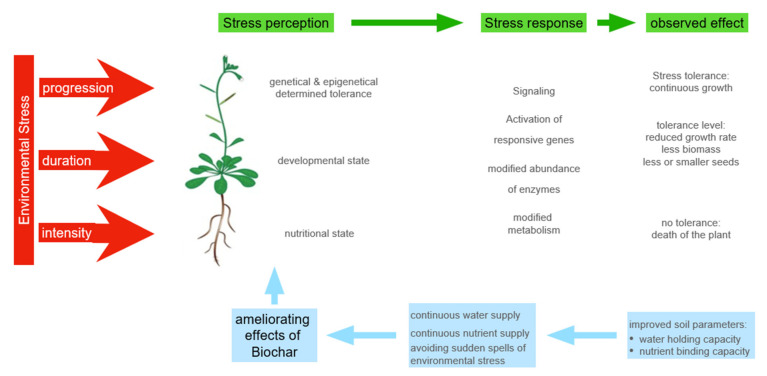
How biochar is improving the stress tolerance of plants.

Therefore, improved plant performance was based on a more even supply of water or nutrients and avoiding extremes (Figure 3). As a result, temporary stressful situations have been largely avoided. Accordingly, the drought and salinity stress tolerance of soybean and rice plants could be significantly improved by the amendment of biochar [122,123]. However, a threshold level of biochar supply was evident [124]. In this case, if the concentration of biochar exceeds this limit, the ion binding capacity in the soil fraction is too high. This creates a competition between the biochar and the roots and limits the availability of nutrients to the plant roots. Moreover, it turned out that the beneficial effects of biochar amendments could be evaluated by measuring the parameters used for monitoring water shortage and nutrient deprivation stress [123]. In summary, biochar primarily improves the reliability of water and nutrient supply, which allows plants to grow efficiently, while the window of stress resistance remains narrow [125].

Additional recent research indicated that the positive effect of biochar is not restricted to the water and nutrient supply to plant roots, but extends to soil microorganisms, as well [121,126]. The beneficial effect of soil inoculation with plant growth promoting bacteria was enhanced by the parallel application of biochar [124]. When measuring the stress-induced inhibition of physiological and biochemical traits, the beneficial effects of biochar and plant growth promoting bacteria were added with all of the tested plant species [127]. This includes mitigation of biotic and abiotic stress effects, as demonstrated in experiments with maize and tomato [128]. Even salt stress resistance of maize improved with the application of biochar, along with plant growth promoting bacteria (*Azotobacter chroococcum* SARS 10 and *Pseudomonas koreensis* MG209738) [127]. In experiments with tomato plants, it was shown that the positive effect includes suppression of pathogen growth, as well [129]. Moreover, the microbial community in the soil of soybean cultures was significantly changed with the application of biochar.

Biochar allows for a continuous supply of water and nutrients. In addition, it prevents the occurrence of sudden extreme events. Plants have more time to adapt and do not need to spend energy on stress responses. Although, in more recent analysis, it was documented that plant–symbiont interaction was supported, as well.

## 5. Interaction of Plants and Microorganisms

### 5.1. General Observations

In the field, plants, fungi, and bacteria form a well-structured community of organisms [130,131,132,133]. The microbial community of plants is called phyto-microbiome [134]. Microorganisms living in the soil, the rhizobiome, are distinguished from endophytes living inside plants. As growth conditions vary to a large degree between individual plant organs, specific populations of endophytic microorganisms can be found in each one [135]. Moreover, with respect to future agricultural application, beneficial microorganisms and pathogens have to be considered separately [136].

**Figure 4:**
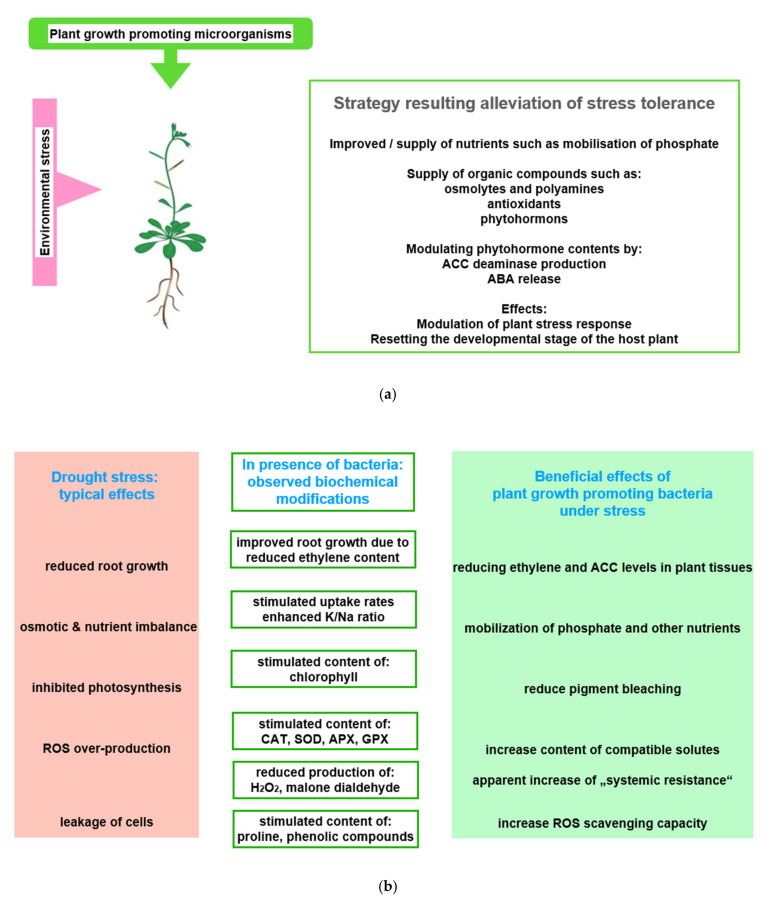

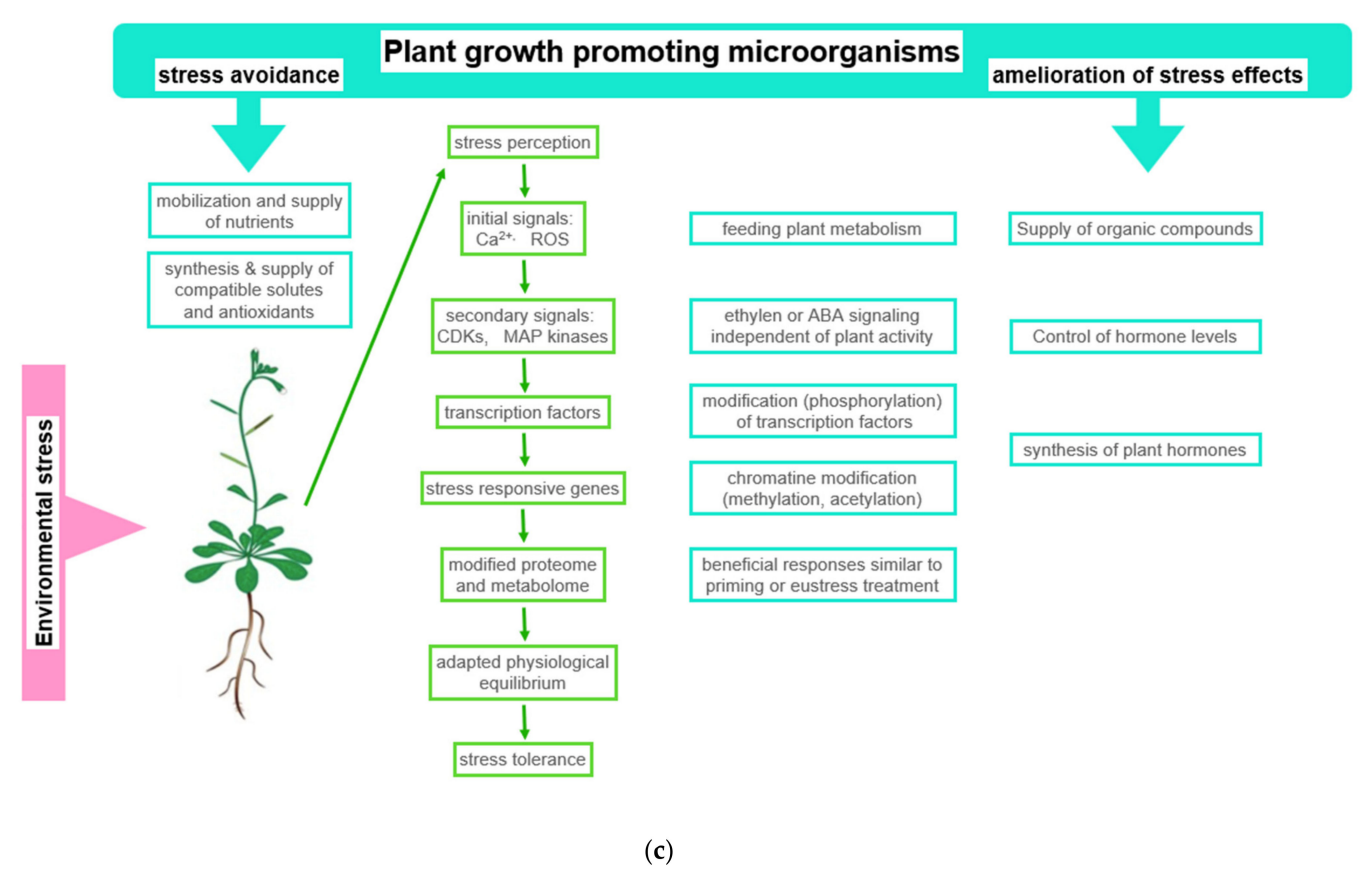
Beneficial effects of plant growth promoting microorganisms (PGPMs). (**a**) Observed stress ameliorating effects of PGPMs: The figure symbolizes the service offered by PGPMs, which helps in hosting plants to improve stress tolerance; (**b**) physiological basis of observed effects: Using drought stress as an example, typical stress effects are listed in the first column. Column two shows how PGPMs modify the biochemical stress response at biochemical level. In column three, the stress responses ameliorated by PGPMs are listed; (**c**) how PGPM activities interfere with the development of plant stress response: The boxes symbolize how PGPM activity may integrate into the reaction sequence, leading to plant stress tolerance. Abbreviations: APX: Ascorbate peroxidase; CAT: Catalase; GPX: Glutathione peroxidase; SOD: Superoxide dismutase.

Research was hampered by the fact that most microorganisms of the phyto-microbiome cannot be cultured in vitro [137,138]. Therefore, information on species numbers and controlling effects, which are exerted on the microbial community by an individual plant genotype as well as the respective developmental stage of plants, was gained only when metagenomic methods became available [139,140,141]. Beneficial effects exerted on plant performance by rhizobia and mycorrhiza were observed early, and turned into a target of investigations [142,143] (Figure 4a). While it was possible to observe rhizobia-induced nodulation and the anatomy of mycorrhiza in the microscope, information on the interaction between plants and their microbial partners were mainly of this indirect type. However, it was found that inoculation of low-fertility soil with plant growth promoting bacteria (PGPB) results in an increased production of biomass, an improved stress resistance, and particularly a reduced sensitivity to incidents of drought stress [59,144,145].

In this context, for instance, the degree of beneficial effects was observed as correlated with the improvement of nitrogen use [146,147]. Recently, in projects that rank the success of inoculation with PGPB, the concentration of intermediates of the TCA cycle, the Calvin–Benson cycle, and photorespiration, which are all extracted from plants proved to be useful parameters and were easily measured in a laboratory [145]. The concentration of these compounds and antioxidants significantly increased in response to osmotic stress [70]. The modulation of these metabolite concentrations, among others, and the alteration of beneficial enzyme activities were interpreted as possible reasons for improved plant growth (Figure 4b). Among the more visible symptoms are enhanced seed germination, an expanded and elongated root system, and an increased chlorophyll content. All of the listed parameters are indicators of plant species-specific mechanism, which relieve the adverse effects of abiotic stress [148,149].

However, data concerning the TCA cycle can sometimes be misinterpreted and misleading. For instance, antioxidants will be degraded under prolonged and intensive stress. However, when monitored over time, the concentration will show a maximum and this maximum will be shifted in the presence of PGPB [67]. While overproduction of these compounds is preferentially achieved by stimulation of plant metabolism, it is assumed that increased concentrations of metabolites of the indol pathway are based on the import of precursors delivered by PGPB. This applies for shikimic, quinic, and salicylic acids [150]. The latter compound functions as a messenger in the hosting plant.

### 5.2. Acquisition of Symbionts

When a plant faces unfavorable conditions, it re-shapes physiological and biochemical parameters, allowing for a modified plant–microbe and microbe–microbe interactions [151,152]. Plants adapt to the patterns and abundance of suitable species of the rhizo-microbiome by the release of various metabolites, such as signaling molecules as well as organic C and N sources for microorganisms to feed on [132,134,151,153,154,155,156,157]. The composition of compounds released by the plants can vary with both the growth conditions and the developmental stage of an individual plant species [158]. The amount of released carbohydrates may resemble 10% of the assimilate production [159]. This explains why the plant rhizosphere contains a significantly higher number of microorganisms as compared with the same soil type in the absence of any plants [160]. The spectrum of microbial species attracted to the plant roots will vary accordingly [161,162,163]. Moreover, it has been observed that the service of the attracted rhizo-microbiome is fine-tuned to the needs of the hosting plant [164,165,166,167,168]. Correspondingly, a modified abundance of microbial species occurs in the root-free soil areas and in samples collected from the root surface, respectively.

In response to compounds released by the plant, microorganisms release signaling compounds and metabolites that are beneficial to plants [158]. This signaling interplay is coordinated between both partners during adaptation to changing environmental conditions [169,170] (Figure 4c). This symbiosis can lead to the segregation of other organisms. Some invasive plant species are capable of modifying the abundance of beneficial microorganisms in the soil in favor of organisms that preferentially support the invading species [168].

### 5.3. Two Examples of Improved Nutrient Supply Provided by Plant Growth Promoting Microorganisms

As previously described, triose phosphates are exported from the chloroplast exclusively in exchange for phosphate. Therefore, a lack of phosphate inhibits the supply of the plant with assimilates, and indirectly prevents the use of absorbed light energy in photosynthesis. Nitrate reduction is performed using reducing equivalents and ATP. The primary product of nitrate assimilation is glutamine. Lack of water and salinity inhibit growth and the need for assimilates. An excess of energy arises, manifested as an over-reduction of the electron transport chain. As a result, a good nitrate supply can relieve the system by discharging ATP and reducing equivalents for nitrate reduction. In fact, it has been found that PGPMs can improve the plant supply of phosphate and nitrate.

#### 5.3.1. Mobilization of Plant-Inaccessible Phosphate

Nutrient supply and use efficiency of crops have received increased attention due to the disappearance of plant available mineral resources and raw material stocks. Therefore, inoculation experiments using plant growth promoting bacteria in combination with conditions for an efficient nutrient use and minimal nutrient losses, were a promising approach to ensure a sustainable nutrient supply. The expectation was an improved nutrient uptake of the plants and growth promoting effects [171,172,173]. The PGPM inoculation leads to changes in the root architecture. This effect was explained by the release of phytohormones, such as IAA to the bacteria hosting plants [174,175,176].

Fertilization with the macronutrient phosphorus is one of the most important strategies to improve crop yield. However, only monobasic (H_2_PO_4_^−^) and dibasic (HPO_4_^2−^) phosphates are resorbed by plant roots (Perez-Montano et al., 2014) and a significant portion of soil phosphorus is unavailable for plants [177,178]. Phosphate solubilization capability of soil microorganisms is an important feature for plant growth enhancement under moderate fertility conditions [177,179,180,181]. Based on their ability to secrete organic acids, acid phosphatase, and alkaline phosphatase, phosphate-solubilizing bacteria are able to lower the pH in the rhizosphere, and in this way, increase the soluble and plant available phosphate fraction [182,183,184]. Notably, as abovementioned, these phosphate-releasing bacteria promote plant growth by offering further compounds in addition to mobile phosphates.

#### 5.3.2. Support of Nitrogen Uptake and Fixation

Few plant growth promoting bacteria species are able to improve nitrogen use efficiency. They are not only involved in N cycling in the rhizosphere, but can also enter plant tissues and modify plant anatomy [185]. Transfer to plant tissues includes incorporation into seeds, thus promoting the development of next generation seedlings [168]. As these bacteria lack the nifH gene, enhanced nitrogen assimilation will occur through mechanisms other than nitrogen fixation [186]. Their bacterial activity correlates with plant species-specific polyamine production [187]. Interestingly, it was observed that the patterns of accumulated polyamines, amino acids, and urea in an experimental plant was modified by the application of stresses, independent of the growth promoting bacteria species present in the test [188,189].

## 6. Response of Plants to Environmental Stress in Dependence of the External Application of Plant Stress Reducing and Growth Promoting Compounds

Exo-polysaccharides, volatile organic compounds, and compatible solutes, such as proline, trehalose, and glycine betaines, were identified as excreted compounds of salt- resistant PGPB species.

Cells of the host plant responded to the application of these bacterial metabolites by the upregulation of a pattern of genes, including the SOS1 gene [190], several stress-responsive genes [191], high-affinity K^+^-transporter genes [192], as well as genes coding for antioxidant scavenging enzymes and ethylene biosynthesis [193]. This concerted regulation of gene activities resulted in an alleviation of salt-stress response and symptoms [194]. Comparable results were obtained with water shortages.

**Figure 5:**
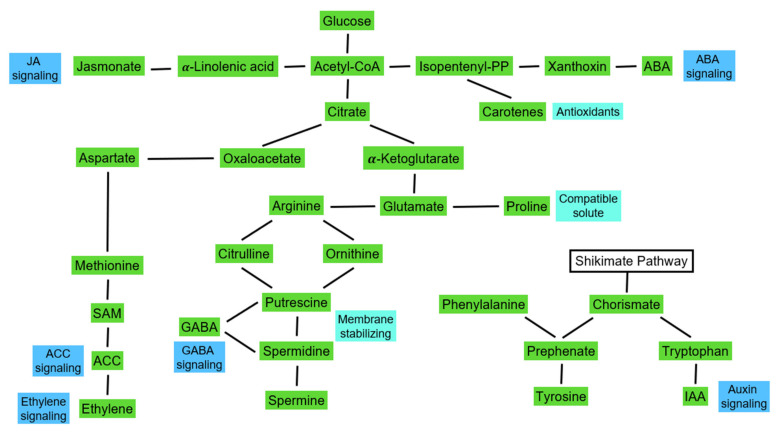
Linking messenger synthesis to primary metabolism. This figure provides a general overview of carbohydrate and amino acid metabolism. It indicates how the synthesis of plant hormones and beneficial compounds is linked to the house-keeping metabolism of plants.

These findings initiated tests to improve the salt resistance of crops with the external application of plant stress reducing and growth promoting compounds. Here, the idea was to counteract incidents of adverse environmental conditions and improve crop yield [195,196,197,198,199,200,201,202]. As expected, the reaction depended on the plant species used due to the following differences: (i) The effect of the applied compound, and (ii) the type of response. The authors explained this variation by differences in both the uptake of the tested compounds and metabolic activity of the tested plant species [203,204]. This latter explanation implies species specific differences in metabolism (Figure 5).

The release of bacterial metabolites is characterized by a high diversity. Most of these compounds, and the corresponding signaling network contribute to the attenuation of stress effects [149,205]. Among these growth promoting and soil improving molecules are plant hormones, such as indole-3-acetic acid (IAA) [206] and other signaling molecules [207], antibiotic preventing pathogen infections [205,208,209,210,211], chelating compounds immobilizing heavy metals [212,213,214,215] or supporting cation uptake, such as siderophores [176,216,217,218,219], as well as organic compounds for metabolization by plants as carbon [177,220] or nitrogen sources [221].

Several interacting signaling networks have been detected in plants [222]. These networks control cellular processes, such as enzyme activation, control of reactive oxygen and nitrogen species, assembly of macromolecules, protein localization and degradation. Moreover, this allows plants to adapt to their respective environment and respond to environmental changes [13,222,223,224]. For example, reactive oxygen species (ROS) and reactive nitrogen species (NOS) are produced and scavenged in all plant cells. Both are important signaling molecules that control meristem activity, flowering time, etc. [223,225,226]. Nevertheless, it is well documented (see above) that ROS production will exceed the ROS scavenging capacity under stress. This detrimental effect of environmental stress is significantly reduced in the presence of growth promoting bacteria [227]. Salicylic acid is involved in the control of ROS production in the cytosol [228]. As the beneficial effect is associated with an increased production of osmolytes, salicylic acid, and jasmonate, as well as a reduction in ethylene production, the signaling sequence resulting in the beneficial effect needs to be elucidated [149,229,230,231,232,233]. Respective investigations are complicated since some of the soil bacteria are capable of producing further plant hormones [234,235,236]. As an example, to provide a general overview, we will discuss below signaling events in the hosting plants at cellular level using ABA and ethylene.

### 6.1. Ethylene and ACC

**Figure 6:**
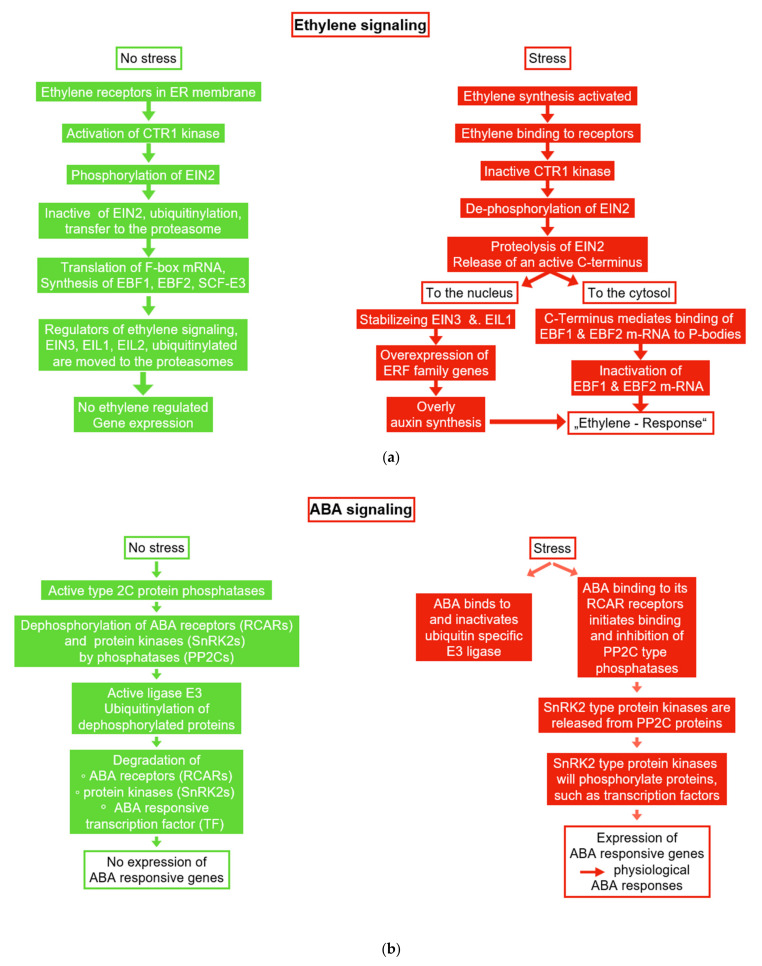
Stress effects on ethylene and ABA signaling. (**a**) Ethylene signaling: The figure indicates how ethylene signaling is modified by stress; (**b**) ABA signaling: The figure indicates how ABA signaling is modified by stress. Abbreviations: EBF1: A transcription factor controlling cell differentiation; EIL1: A transcription factor regulating genes that are responsive to sulfur deficit, for instance; EIN2: A factor involved in histone acetylation; ERF: Transcription factor involved in stress responses; PP2C: Protein phosphatase 2C; PYL: Pyrabactin resistance1/PYR1-like regulator component of the ABA receptor; SCF-E3: Serine ubiquitin ligase; SnRK2: A serine/threonine protein kinase.

In response to environmental stress, plants produce increased amounts of ACC (1-aminoacyclopropane 1-carboxylate) synthase to convert SAM (S-adenosylmethionine) to ACC, the substrate for ethylene synthesis [235] (Figure 6a). Extremely high concentrations of ethylene resulted in inhibition of plant growth [237,238,239]. For this reason, it was not a surprise that the stress-induced synthesis of ethylene was declined in the presence of PGPB [240,241]. A retardation of plant growth by ethylene has been observed as a response to various environmental stresses, such as high salinity [242], drought [243], and the presence of potentially toxic metals [244]. Reduction of this inhibitory effect in the presence of growth promoting microorganisms correlated with a reduction of plant ACC levels and a reduction of the levels of ethylene to non-inhibitory levels [245]. In the genomes of PGPB, the gene coding for the enzyme ACC deaminase was found [244,246,247,248]. For this reason, it may be assumed that ethylene signaling under stress is reduced by a competitive reaction that converts ACC to alpha-ketobutyrate and ammonia [240,249,250,251,252]. Improving plant growth under stress by lowering the ACC content seems to be a common strategy of PGPB, since ACC deaminase has been detected in several bacteria species, such as *Bacillus* spp. [244] and *Pseudomonas* spp. [243,253]. This assumption implies that ACC is not strictly compartmentalized, but forms a pool that extends over plant tissues and PGPBs.

However, recent experiments with Arabidopsis have documented that ACC acts as a signaling molecule and is able to control seedling growth independently of ethylene [254]. This finding was supported by a molecular analysis that correlated with ethylene and ACC-mediated regulation of gene expression in quinoa under hyperosmotic salinity [255]. Based on the general observation that ACC deaminase producing bacteria attenuate adverse effects of long-term stress, such as salinization of soil, it was concluded that in these situations ACC production will exceed its degradation, and its concentration in plants will finally reach a level to inhibit the synthesis of essential metabolites [256].

### 6.2. Abscisic Acid (ABA)

The phytohormone ABA is known for its control of leaf abscission and stomatal closure [257]. ABA synthesis is stimulated by environmental stresses, such as drought and salinity. This results in an adaptive response to conserve water [258]. Moreover, ABA contributes to the regulation of seed germination and fruit ripening [259,260]. Many rhizobacteria have been shown to produce ABA and increase the ABA content of plants [261,262,263,264]. For instance, plant growth promoting rhizobacteria (PGPR) increased the level of ABA in Arabidopsis, tomato, and cotton plants [230,265,266]. The PGPR-inoculated plants showed increased polyamine contents and a lower transpiration rate, but also a reduced plant growth rate. Reduction of leaf MDA content was used as an indicator of reduced ROS production, a measure generally accepted to indicate the degree of stress perception [149]. However, some PGPR can even reduce ABA levels in host plants and indirectly support the increase in plant growth [267] (Figure 6b). This observation indicates that the impact of ABA depends on a signaling network as well as a cross-talk between signaling pathways. For instance, this cross-talk can occur for the following reasons: (i) If protein kinases, reductases or hydrolases are part of a signaling pathway or (ii) signaling molecules, such as Ca^2+^ can target various receptors. In many cases, a cross-talk is observed, since an activated transcription factor can bind to several operon regions of individual stress-responsive genes.

In the case of ABA, the interpretation of experimental results is even more complicated, since the excess ABA concentration can initiate the reduction of metabolite pools (amino acids as well as carotenoids) with high impact on essential functions, such as the chlorophyll synthesis and consequently on photosynthesis and, thus, plant growth rate.

On the other hand, moderate ABA concentrations initiate beneficial reactions of plants, such as improving water use efficiency. Based on these observations, it has been concluded that fine-tuning of the ABA concentration is essential for optimal plant performance in the field [268] and further studies are required to comprehend the interplay with other plant hormones, such as strigolactones [269].

Generally, stress resistance seems to be controlled mostly by the interaction of messengers with transcription factors [270]. As a transcription factor can target several operons, the cascades of controlled genes that react simultaneously explain the observation that regulatory events can simultaneously affect resistance towards multiple stresses [271]. This induction of cross-resistance was analyzed in detail for ABA against several environmental stresses, such as salinity, drought, and temperature. However, detailed information on molecular mechanisms require further investigation, and only a few examples have been identified to date. For instance, it has been observed that TSPO-related sensors (tryptophane-rich sensor proteins) are responsive to salt stress and that water withhold stress. These sensors can be targeted by an external ABA application, as well [272]. In Arabidopsis, two different targets have been identified downstream of TSPO-related proteins: (1) Under stress TSPO-related proteins can stimulate autophagy activity via interaction with autophagy-related protein 8 (ATG8); [273] (2) alternatively, TSPO-related proteins can bind to an aquaporin at the plasma membrane and label it for degradation by autophagy. In this way, ABA stimulation of TSPO-related proteins will reduce intercellular water transport [274].

### 6.3. Regulation of Autophagy by Endophyte and Plant-Induced Signaling under Environmental Stress

Information on stress signaling pathways [13,275,276,277] have been completed by experiments that analyze potential targets [278,279]. Impressive and very detailed studies showed an interplay between de novo synthesis of enzymes and cell structures and degradation of these structures by autophagy [280,281,282]. Autophagy is a subcellular recycling pathway that removes surplus or damaged molecules and organelles. Moreover, it is involved in plant development and stress response [283,284,285]. In a mechanistic sense, the continuous degradation of compounds by autophagy on the one hand, and de novo synthesis of compounds, such as enzymes on the other hand, allows a cell to adapt to varying physiological requirements in a changing environment. This function-centered view makes autophagy a central player in plant stress adaptation and the development of stress resistance. Accordingly, during the last two decades, we can see a continuously increasing number of publications on the physiological and biochemical functions of autophagy and the control of its activity [284,286]. Here, we will focus on the regulation of autophagy by endophyte and plant-induced signaling under environmental stress.

Observed effects of signaling events are interpreted with the assumption of a permanent autophagy potential, but the actual activity is downregulated by inhibitors, such as TOR and COST1 [285,287,288]. During environmental stress, autophagy can be activated by multiple mechanisms, including inactivation of the inhibitors [282,284,285,289,290,291]. These regulatory events are fine-tuned by a signaling network, including the upregulation of gene expression of several transcription factors [284,292,293]. Signal transduction includes post-translational protein modification, such as protein phosphorylation and de-phosphorylation [285,291,294].

To date, the available information makes autophagy a promising candidate for research projects that are aimed at improving crop stress resistance [291,294,295]. While molecular site directed mutation is possible at laboratory scale, the application of results in the field will possibly lack public acceptance in several countries. Taking advantage of recent information, using plant growth promoting microorganisms might be a feasible approach [294].

### 6.4. Control of Autophagy by ABA and Ethylene Signaling

As abovementioned, we have described ABA and ethylene effects as plant stress responses. In addition, we presented that plant growth promoting microorganisms produce ABA, while most of them prevent ethylene production. Moreover, we will continue to focus on these two examples and describe how these two messengers can control autophagy activity.

**Figure 7:**
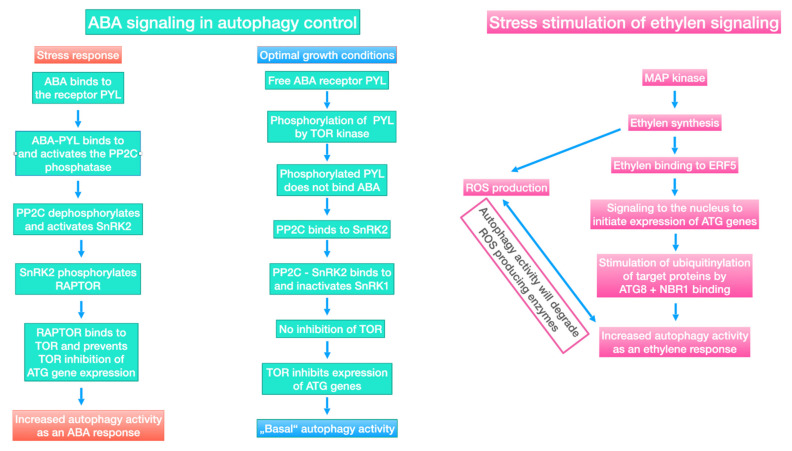
Autophagy control by stress stimulated ABA and ethylene signaling. Autophagy genes can be transcriptionally activated by ABA and ethylene signaling during nutrient starvation as well as environmental stress. On the left, the figure shows how ABA signaling is modified under stress. The right column shows the signaling pathway, leading to ethylene mediated stimulation of autophagy activity. Abbreviations: ATG8: Autophagy-related protein 8; COST1: Constitutively stressed 1, a protein attenuating autophagy by interacting with the autophagy adaptor protein ATG8E; RAPTOR: Regulatory-associated protein of TOR, an adaptor protein; TOR: Target of rapamycin, a serine/threonine protein kinase; TSPO: An 18 kDa translocator protein required to alter the autophagy-lysosomal pathway.

As shown in Figure 7, the damaged molecules and organelles, such as mitochondria, chloroplasts, and peroxisomes are continuously degraded through autophagy [296]. For instance, the resulting breakdown products are used for the de novo synthesis of proteins and organelle biogenesis. As abovementioned, under environmental stress, surplus absorbed light energy may result in an over-production of ROS and subsequent ROS damage (see Figure 2a). In this context, autophagy has two functions: (i) Removal of damaged compounds, and (ii) consumption of surplus energy for de novo synthesis processes. As a result, ROS damage will be repaired, while in parallel, the risk of ROS over-production will be diminished. As a consequence, an improved stress tolerance is observed as the physiological symptom of autophagy activity [290,293,297].

As shown in Figure 7, autophagy is induced by autophagy-related proteins (ATGs) [298,299]. Information on the functions of individual ATGs were summarized in a review by Su et al. (2020) [284]. Regulation of the cellular autophagy activity depends on a network of signaling activities. TOR and COST1 are important players inhibiting autophagy activity [287,288]. Figure 6 shows the examples of ABA and ethylene signaling, particularly how the activity of these inhibitors is controlled under stress. Here, we have focused on both of these signaling pathways to explain the concept that results in the beneficial effects of plant growth promoting microorganisms.

As outlined in several reviews [285,300,301], ATG8 is an ubiquitin-like protein essential for the synthesis of the double-layer membrane, which constitutes the autophagosome vesicle, which is responsible for delivering the cargo from cytoplasm to vacuole lumen. Under normal growth conditions, the protein kinase TOR phosphorylates, the PYL ABA receptors, prevent PYL from binding to ABA and PP2C. SnRK2 and PP2C form repressor complexes, which interact with SnRK1 and prevent it from interacting with TOR (see Figure 7). When the concentration of ABA increases under environmental stress, ABA receptors (PYLs) will bind to ABA and PP2C, inducing the activation of SnRK2. Subsequently, SnRK2 phosphorylates RAPTOR, and in this way, inactivates the TOR complex. As a result of the inhibition of TOR activity, enhanced rates of autophagy can be observed.

ABA producing plant growth promoting microorganisms supply the hosting plants with this hormone. This results in a permanently enhanced ABA level in hosting plants. Among other effects, these plants will show an improved water use efficiency as compared with control plants that do not accommodate the symbionts [245]. Under stress, ABA stimulated autophagy will be of special importance due to the enhanced degradation rates of damaged cell compounds. This will allow for the improved recycling of high value molecules, such as amino acids. Therefore, recycling turns into an important nutrient supply and allows the metabolic activities continue to function [282]. Nevertheless, there will be a plant species-specific threshold level for stress resistance [260,268]. However, up to this level, moderate extra ABA supply will allow hosting plants to grow at a more stable rate.

Inhibition of stress-induced ethylene production is a phenomenon that is quite common to plant endophytes [253,302]. In addition to its effect on plant maturation and fruit ripening, ethylene can inhibit the growth of plant roots [303,304]. Reduced root length and root biomass will enhance the stress sensitivity, particularly with respect to incidents of water shortage and lack of nutrients. Therefore, the beneficial effect of an inhibited ethylene synthesis is evident. In addition to these known effects, there are two signaling pathways that lead to an ethylene-induced stimulation of autophagy activity [305,306]. The question, whether ethylene stimulation of autophagy tends to show an overshoot reaction with the result of major damage of plant tissues, is a matter of current debate [307,308]. It is argued that the inhibition of ethylene synthesis may help the autophagy to remain at a moderate level and prevents a cascade of self-destructing reactions [293,309,310]. In this context, the following have already been shown: (i) Ethylene can activate autophagy via a sequence of reactions involving ROS signaling [311], and (ii) enzymes involved in ROS synthesis are continuously degraded by autophagy activity [312,313]. This regulation loop results in a self-limitation of ethylene-induced autophagy.

### 6.5. Autophagy in Source and Sink Tissues

**Figure 8:**
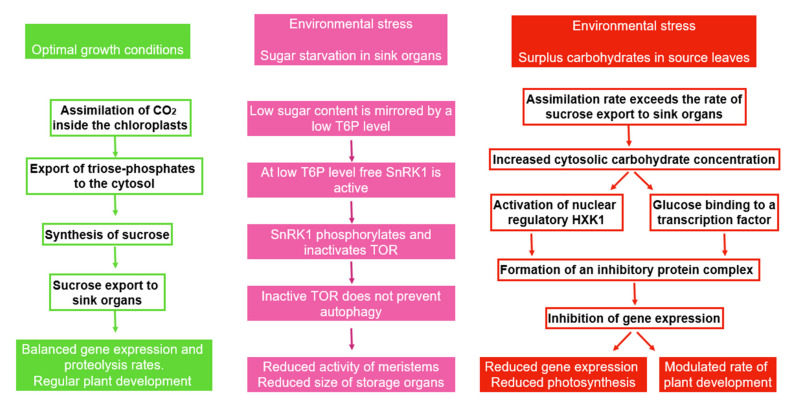
The three columns in this figure indicate how stress may affect the physiological equilibrium in plants. In the left column, the sucrose supply of sink tissues under optimal growth conditions is indicated. The columns in the middle and on the right, respectively, show the modified situation in sink tissues and source leaves under stress.

Carbohydrates, particularly phosphorylated trioses and hexoses, are not only energy sources and substrates of metabolic pathways, but are also important molecules for signaling at cellular level [281,306]. Degradation and recycling of cell components can provide, at least for a limited period of time, the basic building blocks required for the replacement of damaged structures and the adaptation to a stressful situation. Finding a new physiological and biochemical equilibrium is further eased, since the non-essential compounds can be removed and replaced by new essential ones [312]. Experimental analysis of signaling and metabolic sequences requires careful selection and preparation of plant material, since stress-induced sugar accumulation or starvation will initiate different responses in sink and source tissues, respectively [68,314] (Figure 8).

In source leaves, changes in carbohydrate content will regulate the photosynthetic activity of chloroplasts. For instance, stress-induced energy demand in source leaves induces an increased photosynthetic activity and an improved light energy use efficiency [69]. On the other hand, the assimilation rate is downregulated if a stress-induced reduction of sink activity results in an increased sugar content in source leaves. In some plant species, reduction of the rate of assimilation is accompanied with a decrease in CO_2_ fixation through the reduction of the rubisco (ribulose-1,5-bisphosphat-carboxylase-oxygenase) content, while in other species, the rubisco content remains unchanged. Therefore, it can be concluded that downregulation occurs with the regulation of enzyme activity rather than the enzyme content [315].

In soybean, the starvation of carbohydrates in sink tissues results in the expression of a typical pattern of genes, the accumulation of ERF, ACC synthase, and an ethylene-induced transcription factor. It may be expected that these compounds are involved in stimulation of starvation-induced autophagy [305]. There seems to be a correlation between carbohydrate starvation of leaves and induction of autophagy [316].

## 7. Summary

The different types of environmental stress are the major constraints for crop production and food security worldwide [317]. Problems will escalate due to the predicted changes in global climate. Among the environmental stresses, water and nutrient supply have attracted the attention of scientists as well as stakeholders. In principle, there are two options to improve crop yield: (i) Improving the soil’s water and nutrient holding capacity, and (ii) improving the plant’s capability to acquire water and nutrients and to tolerate incidents of inadequate supply [318,319].

The first option requires the linking of structural and functional soil–plant–atmosphere (SPA) interactions and their optimization, as shown in this overview for the soil–plant system. SPA interactions are the focus of agricultural and ecological studies on global environmental change, greenhouse gas emissions, and carbon sequestration across biomes [320]. Responses to environmental stress are often a multifactorial trait [30]. A connection between stress-induced damage and overproduction of ROS has been observed. Therefore, the observed stress effect and the later loss of harvest are based on previous mechanisms. An important reason for this observation is that inhibited growth leads to lower consumption of assimilates, and thus inhibits photosynthesis. As a result, ROS production is an alternative use of absorbed light energy. For this reason, the ROS concentration and ROS-induced damage (the concentration of malondialdehyde) are used by many publications as a measure of the level of stress [321].

Both of the methods described in this review, soil improvement with biochar and inoculation with growth promoting microorganisms, are already used in agriculture. To meet the immediate needs of agriculture more easily and to improve soil–plant interactions, these two methods have already attracted increased attention [116,233,322,323]. Soil improvement with biochar can meet the first requirement, i.e., the enhancement of water and nutrient holding capacity of the soil. On the other hand, the application of PGPB aims at improving the nutrient supply of the plants. Both methods do not have to be understood as alternatives, they can rather complement each other in a meaningful way.

Nevertheless, further research is required to identify the ideal population of microorganisms as symbionts for each individual crop as well as the growing area (it is evident that the populations differ depending on the soil and local climate) [4]. Molecular tools are now available that not only allow for the identification of microbial symbionts, but also the analysis of the contributions of each of the partners in the analyzed symbiosis [324,325,326]. Therefore, it is predictable that both approaches will be established as the mainstream method to mitigate adverse effects of environmental stress on agricultural production [327]. However, the studies need to be extended and the interactions between all parts of the SPA (including the atmosphere) need to be optimized to reduce the bilateral stress of the plants.

New methods in biochemistry and particularly in molecular biology allow the study of the regulation of the plant’s stress response. Tolerant plants will find a new metabolic equilibrium in the presence of stress and will thereafter continue to grow at an adjusted rate. In the second part of the review, we used the example of ABA and ethylene to show how this adaptation can be regulated at the cellular level. In the case of fluctuating environmental conditions in particular, it is important for plants to bridge the phase of adaptation to stress conditions. In this context, we have presented the regulation of autophagy. Autophagy breaks down damaged components of the cells and at the same time provides building blocks (amino acids) for new syntheses [328]. We believe that this function is particularly essential at sites with frequently changing environmental conditions.

## 8. Conclusions

In agricultural breeding, three goals are pursued: (i) Increase in yield through improved adaptation to regional conditions (soil and climate), (ii) improved nutrient use efficiency to save fertilizer and prevent environmental contamination, (iii) resistance to biotic and abiotic stress. To achieve short-term results, targeted breeding approaches are planned. It is well known that the success of new breeds can sometimes not be confirmed at every location. The inclusion of soil conditions, particularly the microbiome, as well as the possible fluctuation of environmental conditions, can offer an explanation. Based on a comprehensive understanding of the interrelationships, new strategies can be developed that include not only the plant, but also the soil and the environmental conditions. A large number of parameters must be taken into account that can only be recorded using data processing methods, such as AI. This problem is illustrated by two examples: (1) For a reproducible use of biochar, it is not sufficient to know the origin of the biomass used. Rather, it must also be stated in which atmosphere the production took place, how quickly it was heated, and which final temperature worked and for how long. (2) Discussions are currently ongoing regarding how to draw conclusions about the population of soil microorganisms. There is often a lack of capacity for a precise laboratory analysis and less than 10% of the species are recorded with standard methods. For practical application, the following points seem to be sufficient: (i) Determine the organic matter content, and (ii) know the previous agricultural use.

The studies presented here open new breeding approaches to improve crop yields. To date, the fluctuation of environmental conditions has only been taken into account to a limited extent. It is known that plants respond to changes in environmental conditions with changes in their physiology and metabolic activity. However, the extent to which autophagy can support the adaptation phase by providing building blocks has not usually been taken into account. At present, autophagy has mainly been seen as a risk that can lead to the destruction of tissues and organs. On the other hand, new breeding approaches are aimed at the improved control of the regulation of these processes. In this context, ROS, which are formed during a stress response, are not only seen as pollutants, but also as messenger substances.

It is evident that targeted breeding using molecular genetic approaches can deliver faster results than classic cross breeding. This applies not only to plants, but also to microorganisms that will be used to inoculate the soil. However, in many countries, GM crops are not accepted by the population. Therefore, alternative methods are applied, in which experiences from laboratory tests are used. The successes achieved through the use of PGPMs on marginal soils show that when discussing the importance of biodiversity, one must think not only of plants, but also of soil microorganisms. Moreover, this indicates that a soil must not only be assessed on the basis of inorganic nutrients, but also on the basis of organic compounds.

## Data Availability

Not applicable.

## Abbreviations

ACC: 1-Aminocyclopropane-1-carboxylate; PGPB: Plant growth promoting bacteria; PGPM: Plant growth promoting microorganisms; PGPR: Plant growth promoting rhizobacteria; ROS: Reactive oxygen species; SOS: Salt overly sensitive; SPA: Soil–plant–atmosphere.
